# Prevalence of *Eimeria* spp. with associated risk factors in dairy calves in Sylhet, Bangladesh

**DOI:** 10.1002/vms3.776

**Published:** 2022-02-24

**Authors:** Liton Chandra Deb, Syed Sayeem Uddin Ahmed, Chandan Chandra Baidhya, Nirmalendu Deb Nath, Sumon Ghosh, Suman Paul

**Affiliations:** ^1^ Department of Population Health and Pathobiology College of Veterinary Medicine North Carolina State University Raleigh North Carolina USA; ^2^ Department of Epidemiology and Public Health Faculty of Veterinary, Animal and Biomedical Sciences Sylhet Agricultural University Sylhet Bangladesh; ^3^ College of Veterinary Medicine University of Tennessee Knoxville Tennessee USA; ^4^ Infectious Diseases Division International Centre for Diarrhoeal Disease Research, Bangladesh (icddr,b) Dhaka Bangladesh

**Keywords:** Bangladesh, calf coccidiosis, calves, Eimeria, prevalence, risk factors

## Abstract

**Background:**

Bovine eimeriosis is thought to be very important for the productivity and health of cattle all over the world. Despite the importance of cattle farming in Sylhet, little is known about the prevalence of bovine *Eimeria* spp. and the risk factors connected with it.

**Objectives:**

We conducted a study to evaluate the prevalence, species diversity and associated risk factors of *Eimeria* spp. in a population of 50 cattle farms from 12 upazilas (sub‐district) in Sylhet district.

**Methods:**

Faecal samples were collected randomly from a total of 554 calves ranging in age from 1 month to 2 years old during a period of 7 months. We used Flotation and McMaster techniques for parasitological examination. Species identification was done by using their morphological and morphometric characteristics.

**Results:**

Out of 554 calves, 308 were found to be positive for *Eimeria* species (55.60%). Seven species of *Eimeria* were identified. Among the identified species, *E. bovis* (38.98%), *E. zuernii* (26.17%) and *E. alabamensis* (22.38%) were found to be the most prevalent species. Mixed and species‐specific *Eimeria* infection were (24.73%; 95% CI 21.32–28.49) and (30.87%; 95% CI 27.17–34.84), respectively. In addition, the highest prevalence was observed at Zakigonj (68%; 95% CI 58.34–76.33) and the lowest at Companygonj (40%; 95% CI 30.94–49.80). *Eimeria* species intensity ranged between 50 and 76,550 oocyst per gram of faeces. Analysis of associated risk factors by using multivariate logistic regression analysis revealed that age, gender and body condition were significantly (*p* < 0.05) associated with *Eimeria* infection.

**Conclusions:**

Based on these present findings, it can be assumed that ‘coccidia belong to the most prevalent pathogens in the population of calves in the study area’. Thus, the findings of this study could be used as tools for adoptive surveillance and effective control and prevention of the disease in cattle populations in this region.

## Graphical abstract

We conducted a study to evaluate the prevalence, species diversity, and associated risk factors of Eimeria spp in Sylhet, Bangladesh. Our study revealed that age, gender, and body condition were significantly associated with Eimeria infection indicating high prevalence of coccidial pathogens in the population of calves in the study area. The findings of this study could be used as tools for adoptive surveillance and effective control and prevention of the disease in cattle populations in this region.

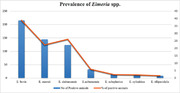

## 1 INTRODUCTION

Protozoan diseases are a limitation for the advancement of dairy farming globally, especially in developing nations (Farooq et al., 2012; Om et al., 2010). Bovine coccidiosis is an important protozoan infection all over the world caused by species of the genus *Eimeria* in cattle, and it causes significant economic losses every year for the meat and farm businesses (Daugschies & Najdrowski, 2005). It exerts enormous economic losses to livestock industries through decreasing productivity such as reduced appetite, weight loss, reduction in growth rate, a dull appearance, and also, morbidity, mortality, the expense of treatment, and control measures (Nalbantoglu et al., 2008). More than 12 species of *Eimeria* in cattle have been documented so far. The predominant species are *E*. *bovis*, *E*. *zuernii*, *E*. *auburnensis*, *E*. *canadensis*, *E*. *ellipsoidalis*, *E*. *subspherica*, *E. cylindrica*, *E*. *alabamensis*, *E*. *wyomingensis*, *E*. *bukidnonensis*, *E*. *illinoisensis*, and *E*. *brasilensis* (Das et al., 2015; Dong et al., 2012; Kim et al., 2018). Generally, the most pervasive species are *E*. *bovis*, *E*. *zuernii*, *E*. *ellipsoidalis*, and *E*. *auburnensis* (Das et al., 2015; Dong et al., 2012).

Coccidiosis is an intestinal disease that affects all ages of cattle; however, clinical coccidiosis is most common in young animals, especially calves. It mainly occurs particularly because immunity is not well developed at a young age. It is frequently observed under conditions of intensive feeding and management systems (Rehman et al., 2011). Calf coccidiosis usually occurs as a subclinical disease with no apparent signs of infection. It involves great financial losses attributable to decreased appetite, weight loss, feed conversion rates, unthriftiness, diarrhoea, dysentery, anaemia, and increased susceptibility to other diseases (Bohrmann, 1991; Daugschies & Najdrowski, 2005).

The prevalence of *Eimeria* in cattle and buffaloes has been well reported from across the world (Dong et al., 2012; Khan et al., 2013; Laha et al., 2013), including Bangladesh (Samad et al., 2004), but information regarding the prevalence of calf coccidiosis in Bangladesh is very scarce. One study on calf morbidity and mortality in Bangladesh reported a prevalence of 27% for calf coccidiosis (Samad et al., 2001). Despite its known effect on calf morbidity and mortality, no epidemiological study has focused on calf coccidiosis in Bangladesh. Thus, information on the epidemiology of calf coccidiosis remains scarce.

Sylhet, a north‐eastern district of Bangladesh, has 6 to 7 million (BBS, 2011) of cattle, mostly composed of indigenous non‐descriptive cattle reared in a small‐scale semi‐intensive system. Calf mortality was reported to be very high in this area, and a considerable portion of this mortality was attributed to gastrointestinal infection. However, based on the pattern and clinical symptoms, a considerable portion of this mortality could be due to coccidia infection. Thus, an epidemiological study was performed to explore the status of calf coccidiosis and to identify different *Eimeria* species that cause coccidiosis considering possible contributing risk factors in calves in Sylhet district of Bangladesh.

## 2 MATERIALS AND METHODS

### 2.1 Study area

The study was conducted from June to December 2019 in all 12 upazilas (sub‐districts) of Sylhet district. Sylhet district is situated in the northeastern region of Bangladesh (24.8917° North latitude and 91.8833° East longitude). It occupies an area of 3452 km^2^. The climate of Sylhet is humid subtropical with an annual average temperature of 24°C and an annual average rainfall of 333 mm.

### 2.2 Reference and study population

Calves from household and commercial farms in the Sylhet district of Bangladesh were the reference population. A list of the farms was collected from the local Upazila Livestock Offices as a sampling frame; five farms from each upazila were selected from this list using simple random sampling methods. Farms (*N* = 50) having at least 5 calves ≤ 2 years old, were selected to be sampled in this study. Therefore, a total of 554 calves were sampled, as per set criteria as follows: if there were 5–10 calves/farm, then all were sampled. If there were >10 calves, then 10 calves were selected by simple random.

### 2.3 Collection of faecal samples

Total 554 faecal samples of individual calves under 2 years of age from 50 cattle farms in Sylhet district were rectally collected. Each faecal sample was placed in a clean, sterile plastic container marked with identification numbers and then immediately transferred to the Medicine Laboratory of Sylhet Agricultural University (SAU) and stored at 4°C.

### 2.4 Data collection

**FIGURE 1:**
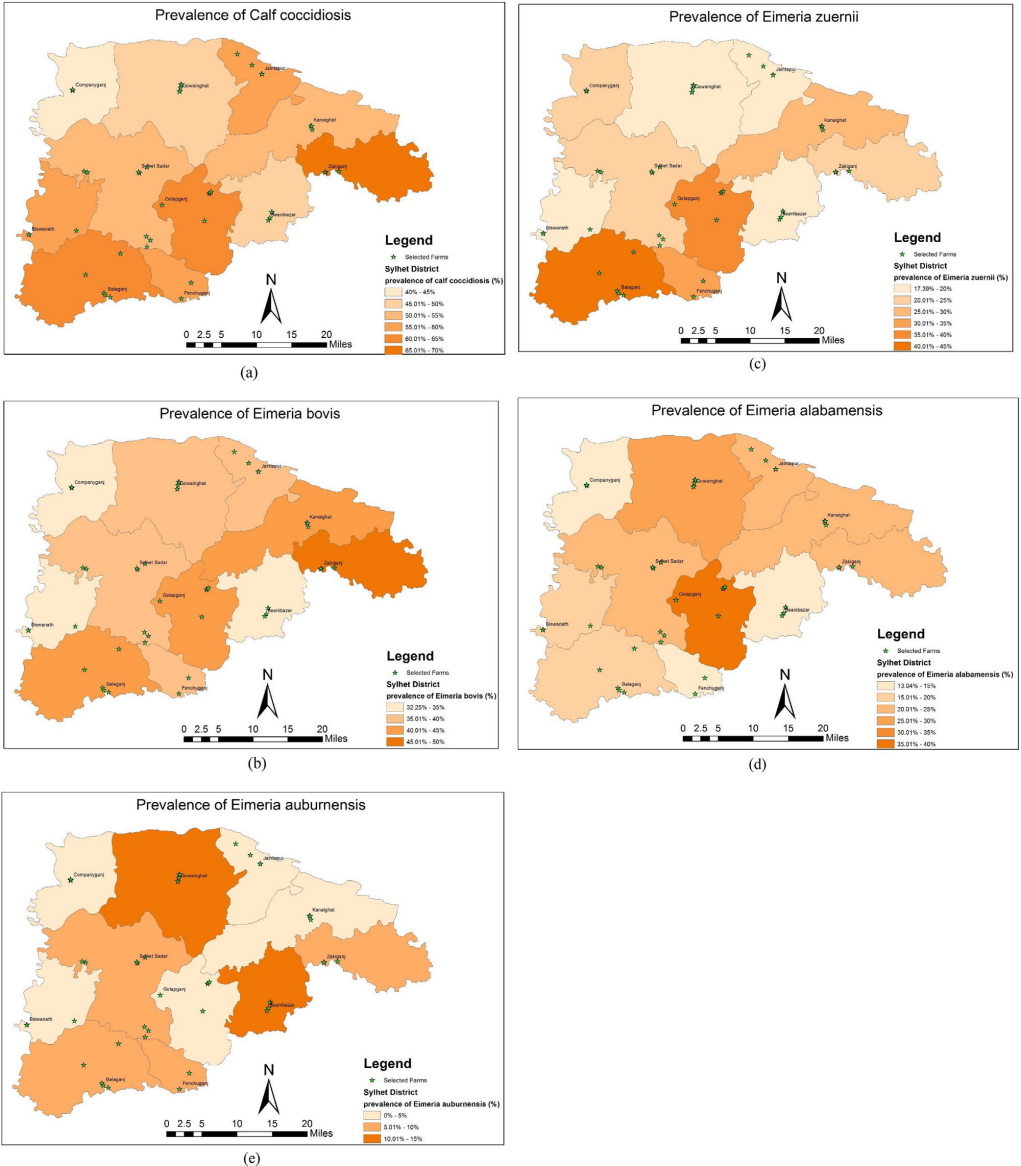
Geographical distributions of the prevalence of overall and species‐specific coccidial infection identified in calves of sampled farms in Sylhet district during (*n* = 554 calves; *N* = 50 farms): (a) prevalence of calf coccidiosis, (b) prevalence of *Eimeria bovis*, (c) prevalence of *Eimeria zuernii*, (d) prevalence of *Eimeria alabamensis*, and (e) prevalence of *Eimeria auburnensis*

The individual animal information and farm‐level information were collected from the farmer's record books and the face‐to‐face interview of the farmers of the selected farms using a well‐designed pretested questionnaire. The geographical information of each of these selected farms was captured as a geographical coordinate by Global Positioning (GPS) Reader (see Figure 1 for exact locations).

### 2.5 Examination of faecal sample

All collected samples were examined qualitatively, and quantitatively with routine copro parasitological techniques. Flotation method with saturated NaCl was used to identify the presence of *Eimeria* oocysts. The procedure was adopted as described by (Zajac & Conboy, 2012). A slightly modified form of the McMaster technique described by Soulsby (1968) was performed to determine the oocysts per gram of faeces (OPG). If 1 g faeces of a calf contained ≥50 oocysts of any species of *Eimeria*, that is, at least one oocyst of any species of *Eimeria* in McMaster chamber, which is equivalent to ≥50 OPG, then the calf was considered to be positive, otherwise negative (Alemayehu et al., 2013). Identification of *Eimeria* species was based on the morphological features of the oocysts such as size, shape, colour, presence or absence of micropyle, polar cap, and so on, with the aid of taxonomic keys (Rehman et al., 2011; Soulsby, 1968). The size of the oocysts was measured by using an ocular micrometre.

### 2.6 Statistical analysis

Prevalence was calculated as the proportion of positive calves among the sampled individuals. The precision of this estimate was ensured by calculating the 95% confidence interval of the proportion. Association between the prevalence of coccidiosis and different intrinsic/extrinsic factors was evaluated by chi square (*χ* ^2^ test). Independent associations between the various factors and coccidiosis were assessed using logistic analysis with a backward elimination process. A *p*‐value < 0.05 was considered statistically significant. Statistical Analysis System (SAS) version 9.4 was used to perform all statistical analyses.

### 2.7 Disease mapping

Geo‐spatial data (latitude and longitude coordinates of selected farms) along with farm ID were collected for each study site, and then entered and stored in a Microsoft Excel spreadsheet. Prevalence maps of overall and species‐specific (single) infection of calf coccidiosis were constructed in ArcMap 10.1 and ArCatalog 10.1.

## 3 RESULTS

The overall prevalence of calf coccidiosis in Sylhet was (55.60%; 95% CI 51.44–59.68). Prevalences of mixed *Eimeria* and species‐specific *Eimeria* infections were (24.73%; 95% CI 21.32–28.49) and (30.87%; 95% CI 27.17–34.84), respectively (Table 1). In addition, a high prevalence of *Eimeria* infection was estimated across the study upazilas (68%–40%). Among the upazilas, the highest prevalence of coccidiosis (68%; 95% CI 58.34–76.33) was observed at Zakiganj and the lowest prevalence (40%; 95%CI 30.94–49.80) at Companyganj. The geographical distributions of the prevalence of overall and species‐specific *Eimeria* infection in Sylhet district were presented in Figure 1.

**TABLE 1:** Prevalence of overall, mixed and single infection of calf coccidiosis in Sylhet, Bangladesh, 2019

Infection type	No of positive animals	% of positive animals (95%CI)
Mixed infection	137	24.73 (21.32–28.49)
Single infection	171	30.87 (27.02–34.72)
*E. bovis*	89	16.06 (13.24–19.35)
*E. alabamensis*	37	6.68 (4.89–9.07)
*E. zuernii*	41	7.40 (5.50–9.89)
*E. auburmensis*	4	0.72 (0.28–1.84)
Total	308	55.60 (51.44–59.68)

**FIGURE 2:**
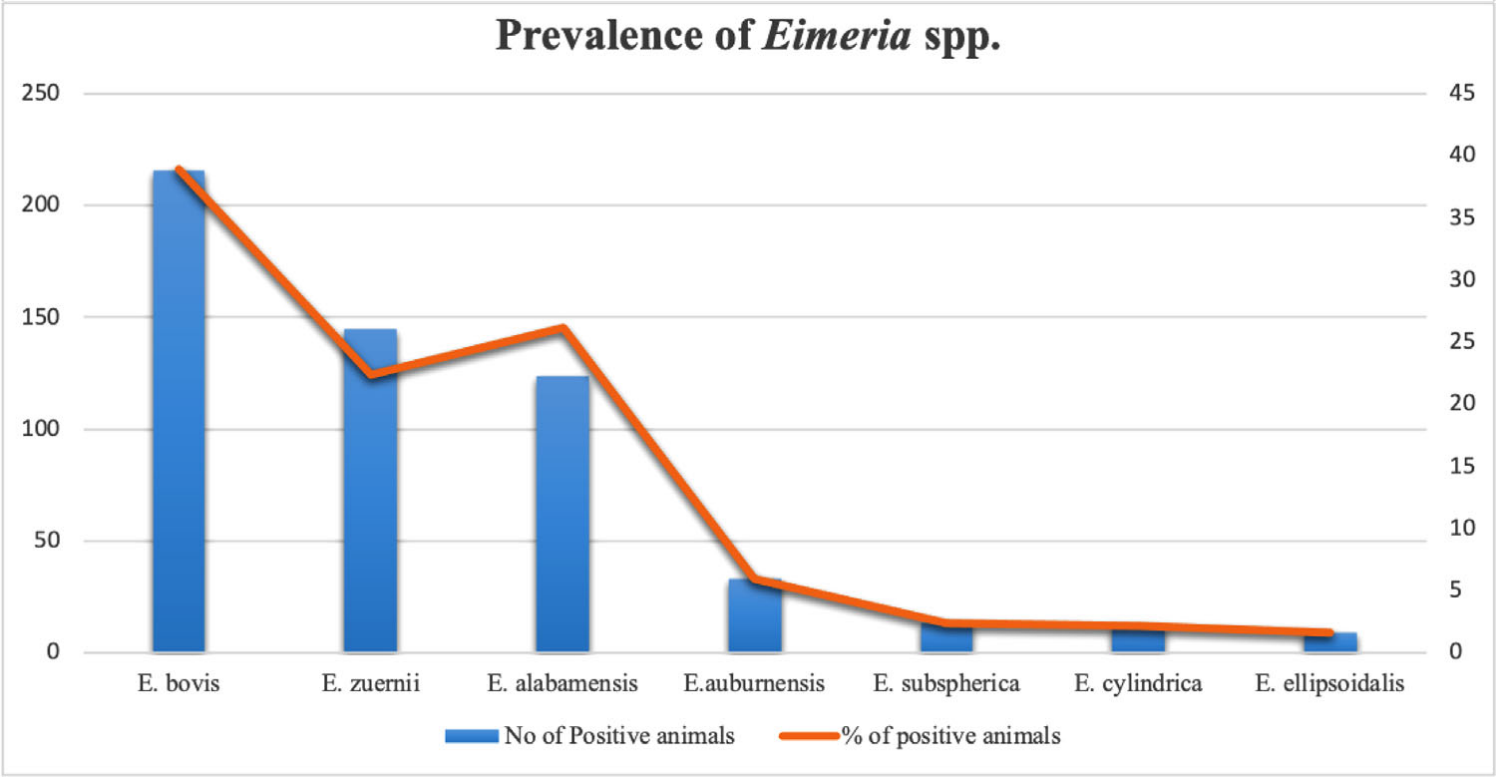
Prevalence of different *Eimeria* spp. identified in calves of sampled farms at Sylhet, Bangladesh

Seven *Eimeria* species were identified among 308 *Eimeria*‐infected samples (Figure 2). The most prevalent species were *E. bovis* (16.06%), followed by *E. zuernii* (7.4%) and *E. alabamensis* (6.68%) (Table 1). Concurrent infections with more than one *Eimeria* species were very common (Table 2). Not more than 5 *Eimeria* spp. were involved simultaneously in an infection; 44.47% of positive calves carried two to five species. Mixed infections involving two to three species were observed in 38.31% of the positive samples.

**TABLE 2:** The number of Eimeria spp. in individual positive faecal samples of cattle (*n* = 308) in Sylhet, Bangladesh, 2019

No. of Eimeria spp.	No of positive samples	% of positive samples (95% CI)
1	171	55.52 (49.80–61.20)
2	51	16.56 (12.60–21.20)
3	67	21.75 (17.30–26.80)
4	18	5.84 (3.50–9.10)
5	1	0.32 (0.0–1.80)

Analysis of all assumed risk factors by chi‐square and stepwise multivariate logistic regression model with backward elimination revealed that age, gender and body condition were the factors significantly associated (*p* < 0.05) with *Eimeria* infection (Table 3 & 4). Young calves (≤6 months) had a significantly higher prevalence (OR = 1.98; 95% CI 1.36–2.82) than young cattle (>6 months). A strong association (*p* < 0.05) was observed between gender and risk of *Eimeria* infection. *Eimeria* infection was more prevalent in female calves (OR = 1.91; 95% CI 1.35–2.72) as compared to male calves (Table 4). There was a statistically significant association (*p* < 0.05) observed between body condition and prevalence of *Eimeria* infection. The results revealed that *Eimeria* infection was more prevalent in calves with poor body condition (OR = 1.66; 95% CI 1.15–2.39) compared to calves having good body condition (Table 4).

**TABLE 3:** Univariable analysis (chi‐square test) of plausible determinants of calf coccidiosis in Sylhet, Bangladesh, 2019

Variables	Total number of study population (*N*)	Number of positive (*n*)	Prevalence (%)	Chi‐square value (*p*‐value)
Age				15.2990 (<0.0001)
≤6 months	231	151	65.37	
>6 months	323	157	48.61	
Herd size				0.4517 (0.5011)
Larger	192	103	53.65	
Smaller	362	205	56.63	
Gender				8.3629 (0.0038)
Male	257	126	49.03	
Female	297	182	61.28	
Area				1.2548 (0.2622)
Hilly	276	160	57.97	
Plain	278	148	53.24	
Breed				0.4979 (0.4800)
Cross	297	161	54.21	
Local	257	147	57.20	
Body condition				10.2848 (0.0013)
Good	209	98	46.89	
Poor	345	210	60.87	
Water				1.1855 (0.2758)
Pond	351	189	53.85	
Deep tubewel	203	119	58.62	
Housing				0.7337 (0.3913)
Intensive	250	134	53.60	
Semi‐intensive	304	174	57.24	
Floor type				0.0694 (0.7920)
Cemented	357	197	55.18	
Non‐cemented	197	111	56.35	

**TABLE 4:** Final model of multivariable logistic regression analysis of plausible determinants of calf coccidiosis in Sylhet, Bangladesh, 2019

Variables	Odds ratio (95% Confidence interval)	*p*
Age		0.0003
≤6 months	1.98 (1.36–2.82)	
>6 months	1	
Gender		0.0148
Female	1.91 (1.35–2.72)	
Male	1	
Body Condition		0.0062
Poor	1.66 (1.15–2.39)	
Good	1	

## 4 DISCUSSION

Epidemiological evidence on diseases is essential for disease management and an adoptive control programme (Bonneux & van Damme, 2011). Calf coccidiosis is considered an endemic disease worldwide, and its existence has been reported in Bangladesh (Nath et al., 2013; Samad et al., 2004). However, information on epidemiological indices such as prevalence and determinants of this disease based on field observations was minimal. Thus, field‐based epidemiological research on calf coccidiosis is deemed necessary to assess the magnitude and to explore the epidemiology of this disease.

This study confirms a high overall prevalence (55.60%) of calf coccidiosis at Sylhet District in Bangladesh. This outcome was not an unexpected result as calf coccidiosis is considered one of the most prevailing and frequently occurring parasitic diseases in young calves (Wood et al., 2013). This prevalence is higher than the prevalence reported in two earlier studies at Mymensingh and Chittagong (Nath et al., 2013; Samad et al., 2001). Geo‐ecological differences along with the difference in study design could explain the differences in prevalence estimates of this study with these two earlier studies. Sylhet is a district with high rainfall and most cattle in this area are raised in a traditional semi‐intensive system where cattle graze on damp marshland. This might contribute to the high prevalence of calf coccidiosis in this area compared to other parts of Bangladesh. Prevalence estimates reported from other tropical and subtropical neighbouring countries like India (Kaur & Kaur, 2008), Pakistan (Rehman et al., 2011) and China (Dong et al., 2012) were 50%, 47.09%, and 47.1%, respectively, and thus, at a prevalence level close to the values found in the present study. A similar result was also reported from Ethiopia (Regasa et al., 2017) with 48.4% prevalence. Contrary to that, lower prevalence estimates from the estimate of this present study were reported from Korea (Kim et al., 2018) as 25.9%. This variation was most likely attributed due to different agro‐ecology, climate, geography, management systems, breeds, and husbandry practices of the examined animals in different countries.

The findings of this study showed that the odds of *Eimeria* infection were 1.98 times higher among young calves (≤6 months) compared with cattle (> 6 months) of age. This finding is supported by results from other studies, showing that *Eimeria* infection is observed more frequently in young calves. A similar age correlation with *Eimeria* infection was reported in a study on the Pakistan cattle populations where *Eimeria* prevalence in calves is higher than in adults (Rehman et al., 2011). Dong et al. (2012) also found the highest prevalence in calves younger than 4 months and lowest in animals of 12 months or older. The higher infection rate in calves is probably due to immature immunity.

Estimated prevalence in crossbred calves did not significantly differ from the prevalence in local indigenous calves, indicating that unlike most other infections and parasitic diseases, breed differences might not play an important role in the occurrence of calf coccidiosis. The results of this current study showed that the risk of *Eimeria* infection in female calves was higher than in male calves. Similar results were reported by Rehman et al. (2011) from Toba Tek Singh, Pakistan. The reasons for this gender variation are unknown. A possible explanation is that young bulls are mostly used for fattening purposes and kept under better management and hygienic conditions than the heifers.

Strong association of *Eimeria* infection with the body condition of cattle has been demonstrated in this study. Calves having poor body conditions showed significantly higher odds of *Eimeria* infection than calves having good body condition. Body condition demonstrates the nutritional status of the calf. Poor body condition of a calf indicates malnourishment, due to the imbalanced feed, which can result in several conditions like ill health and low immunity against infection. This finding is in line with other published investigations, including the study in Ethiopia by Nuriye et al. (2016) that reported body condition correlated with *Eimeria* infection and that the rate of infection was higher in cattle with poor body condition.

The present study identified seven *Eimeria* spp. from the collected samples, namely *E. bovis*, *E. zuernii*, *E. alabamensis*, *E. auburnensis*, *E. ellipsoidalis*, *E. subspherica* and *E. cylindrica* (Figure 2). Farkas et al. (2007) identified the same seven species of *Eimeria* from calves in Hungary. However, Gupta et al. (2016) from Punjab, India, Koutny et al. (2012) from Austria, Heidari et al. (2014) from Iran and El‐Seify et al. (2012) from Kafr el‐Sheikh reported more species of *Eimeria* than found in the present study. On the other hand, the number of *Eimeria* species reported (Alemayehu et al., 2013) from South wollo, Ethiopia and (Rehman et al., 2011) from Toba Tek Singh, Pakistan, were less than the species recorded in the present study. These data indicate that the number of *Eimeria* species varies globally and probably depends on geo‐climatic conditions, management and husbandry. All earlier studies on calf coccidiosis in Bangladesh focused on estimating overall prevalence and *Eimeria* species were not identified or reported (Nath et al., 2013; Samad et al., 2001). Also, there are no documented sensitivity and specificity estimates of the NaCL and McMaster tests in the context of Bangladesh. Thus, we relied on apparent estimates.

*E. bovis* was the most prevalent (38.98%) of species recorded in the calf faecal samples followed by *E. zuernii* (26.17%), and *E. alabamensis* (22.38%) (Figure 2). These three species are considered the most prevailing pathogenic species among all currently known *Eimeria* species occurring worldwide (Alemayehu et al., 2013; Dong et al., 2012). This outcome confirms that all relevant pathogenic species of *Eimeria* are present in the study area and might produce clinical coccidiosis that remained unnoticed or misclassified as other enteric diseases. Mixed infections caused by two to three species were found in 38.31 % of cases, and the number of *Eimeria* species per sample examined in mixed infections ranged from 2 to 5.

Prevalence maps presented in Figure 1(a) indicate that calf coccidiosis is present in all upazilas of Sylhet district with varying prevalence. The highest prevalence of coccidiosis in general and prevalence of *E. bovis* in particular was observed in Zakiganj upazila. On the other hand, Balagonj had the highest prevalence of *E. Zuernii*, and at Golapgonj upazila, prevalence of *E. alabamensis* was the highest. Importance of the infection was not investigated. For this, clinical and/or economic data would have to be collected and analysed.

## 5 CONCLUSIONS

This study revealed a high prevalence of *Eimeria* infection in calves in Sylhet district of Bangladesh (55.60%). Further, the study identified seven *Eimeria* spp.: *E. bovis, E. zuernii, E. auburnensis, E. ellipsoidalis, E. subspherica, E. cylindrica*, and *E. alabamensis*. Among all the identified species, *E. bovis, E. alabamensis*, and *E. zuernii* were the most relevant pathogens in terms of disease risk. Based on multivariate analyses, it was identified that younger calves and females were more likely to be infected than young cattle and male, respectively. The present status of coccidiosis in calves is alarming for cattle farming in the study area. Further epidemiological investigation on coccidian species, other ecological and environmental factors and economic analysis of the effect of the disease is needed to explore the complete epidemiology and ecology of calf coccidiosis.

## ETHICS STATEMENT

This study was conducted according to the instruction and approval of the Department of Epidemiology and Public Health, Sylhet Agricultural University, Bangladesh.

## AUTHOR CONTRIBUTIONS

**Liton Chandra Deb**: Conceptualization, Data curation, Formal analysis, Methodology, Software, Validation, Writing ‐ original draft; **Syed Sayeem Uddin Ahmed**: Investigation, Software, Supervision, Validation, Visualization, Writing review & editing; **Chandan chandra Baidhya**: Conceptualization, Investigation, Project administration, Software, Writing review & editing; **Nirmalendu Deb Nath**: Conceptualization, Software, Visualization, Writing review & editing; **Sumon Ghosh**: Methodology, Supervision, Validation, Visualization, Writing review & editing; **Suman Paul**: Conceptualization, Investigation, Supervision, Validation, Writing review & editing

### PEER REVIEW

The peer review history for this article is available at https://publons.com/publon/10.1002/vms3.776.

## Data Availability

The data that support the findings of this study are available from the corresponding author upon reasonable request.
